# Consumers’ Intentions to Adopt Blockchain-Based Personal Health Records and Data Sharing: Focus Group Study

**DOI:** 10.2196/21995

**Published:** 2020-11-05

**Authors:** Chang Lu, Danielle Batista, Hoda Hamouda, Victoria Lemieux

**Affiliations:** 1 Blockchain@UBC University of British Columbia Vancouver, BC Canada; 2 School of Information University of British Columbia Vancouver, BC Canada

**Keywords:** blockchain, personal health record, health data sharing, consumers’ intentions to adopt, focus group study, microinterlocutor analysis

## Abstract

**Background:**

Although researchers are giving increased attention to blockchain-based personal health records (PHRs) and data sharing, the majority of research focuses on technical design. Very little is known about health care consumers’ intentions to adopt the applications.

**Objective:**

This study aims to explore the intentions and concerns of health care consumers regarding the adoption of blockchain-based personal health records and data sharing.

**Methods:**

Three focus groups were conducted, in which 26 participants were shown a prototype of a user interface for a self-sovereign blockchain-based PHR system (ie, a system in which the individual owns, has custody of, and controls access to their personal health information) to be used for privacy and secure health data sharing. A microinterlocutor analysis of focus group transcriptions was performed to show a descriptive overview of participant responses. NVivo 12.0 was used to code the categories of the responses.

**Results:**

Participants did not exhibit a substantial increase in their willingness to become owners of health data and share the data with third parties after the blockchain solution was introduced. Participants were concerned about the risks of losing private keys, the resulting difficulty in accessing care, and the irrevocability of data access on blockchain. They did, however, favor a blockchain-based PHR that incorporates a private key recovery system and offers a health wallet hosted by government or other positively perceived organizations. They were more inclined to share data via blockchain if the third party used the data for collective good and offered participants nonmonetary forms of compensation and if the access could be revoked from the third party.

**Conclusions:**

Health care consumers were not strongly inclined to adopt blockchain-based PHRs and health data sharing. However, their intentions may increase when the concerns and recommendations demonstrated in this study are considered in application design.

## Introduction

Recently, researchers have suggested that blockchain technology may precipitate a paradigm shift in personal health records (PHRs) [1-4]. A PHR is “an electronic application through which individuals can access, manage, and share their health information, and that of others for whom they are authorized, in a private, secure, and confidential environment” [5], and blockchain refers to “a distributed ledger—write once and never erase” [2]. Current PHR systems commonly use the traditional centralized in-memory or cloud-based database technologies and are designed in such a way that health care providers and organizations continue to be the key controllers of health records despite the intention to engage consumers in managing health care information [6-8]. In other words, it is physicians and health administrators rather than consumers or patients that determine the usage and distribution of health data. Such systems expose consumers to the “single point of failure” of traditional database technologies, in which the information of numerous patients could be lost or altered if the central database is attacked. These systems are also limited in their capability of transferring the ownership of health data to individuals, since the design and maintenance of such systems heavily rely on the resources of organizations, and individuals are not provided with any incentives to be active owners of their data [6,7,9]. As a result, the systems become the barriers to the long called-for patient-centered care [10-12]. By contrast, blockchain is a decentralized ledger where data storage, validation, and synchronization can only be completed when all the contributing system participants (ie, nodes) contribute their computational capacity. As such, a blockchain-based PHR system has the potential to enable health care consumers to take control of their health information, paving the way to materializing patient-centered care [1-4,13]. In addition, as blockchain combines cryptography, peer-to-peer networking, and the Merkle tree structure, blockchain-based PHRs can significantly decrease the risk of data breach, falsification, and tampering, protecting data security and privacy to the level that existing PHR systems are unable to reach [1,2,14]. In addition, blockchain has the technical capability of automatically synchronizing data with all the participating nodes on the network; hence, blockchain-based PHRs can surpass the existing PHR systems in addressing the problem of data siloing and fragmentation [2,15].

Equipping PHRs with blockchain also provides individuals the opportunity to share health data at their discretion with interested third parties, while privacy and security risks are minimized [16,17]. Following prior studies [18,19], this paper uses the terms “individual,” “patient,” and “consumer” interchangeably, since “PHR system consumers are not necessarily dealing with immediate medical concerns and can be ill or healthy” [18]. We refer to third parties as any stakeholders that are outside the patient-physician dyadic relationship while holding interest in individuals’ health information, such as medical researchers, pharmaceutical companies, insurance companies, and employers. These third parties, as important components of the health care ecosystem, have long been using patient data in key organizational activities but have been reported to be collecting and exploiting the data without patient consent or sharing the data in a way that jeopardizes patient privacy [20-23]. Although studies have shown that individuals are generally not opposed to data sharing with these third parties [20,21], the concerns about privacy and security are growing, and individuals demand greater transparency as to who can access their data after they are shared and for what purpose the data are used [22,23]. Since blockchain can combine cryptography with smart contracts, smart contracts being small bits of code that embed procedural logic that is automatically executable, blockchain-based PHRs can ensure that it is consumers who initiate data sharing. This capability allows consumers to determine which data to share with which third party and under what conditions [2,16], preserving their privacy and their right to be informed to an unparallel degree. In addition, smart contracts can be combined with data tokenization, the transformation through cryptography of data into discrete objects that can be transferred over a blockchain network. Besides combining smart contracts with data tokenization, a blockchain-based PHR system can offer consumers the possibility of being rewarded for sharing health data; as such, they can be more incentivized to actively share data with third parties, such as researchers and insurance companies, which can greatly benefit medical research, drug discovery, and care management [24,25]. Specifically, with blockchain, individuals hold a health wallet, in which particular types of health data are stored, and they are the only ones that know the private key needed to access the wallet. When needed, they can share the data with third parties under the conditions specified by the smart contract, such as sharing particular pieces of the data (eg, a biomarker measure) and fees for usage. The data can be tokenized and the third parties may pay for the data use with cryptocurrency or other value tokens.

Recognizing the advantages of blockchain-based PHRs and health data sharing, researchers have begun to give focused attention to blockchain architecture and platform selection [1,4,17]. Moreover, distributed health networks and key health care organizations, such as the University Health Network and Mayo Clinic, have launched pilot applications [26,27]. However, both academics and practitioners have mainly focused on the technical components of blockchain-based PHRs and health data sharing and have conducted these studies from the vantage point of care providers. Very little is known about individuals’ intentions to adopt the blockchain-based system, specifically whether they perceive themselves as motivated to and capable of taking greater control of their health information by using blockchain-based PHRs, whether they are willing to share data when blockchain is incorporated into the system, what concerns they may have about blockchain-based PHRs and health data sharing, and how these concerns can be addressed to enhance their intention. Understanding these questions can not only contribute to the understanding of consumers’ receptiveness to blockchain-based health systems broadly but also inform researchers and practitioners about the issues that they are likely to encounter when designing a blockchain-based PHR system, allowing them to improve the user centeredness of the design [28]. For these reasons, we conducted a focus group study to uncover consumers’ intentions to adopt blockchain-based PHRs and health data sharing.

## Methods

### Overview

We conducted 3 focus groups to understand consumers’ intentions to adopt blockchain-based PHRs and health data sharing, observing a semistructured list of questions (Multimedia Appendix 1). Focus groups are suitable for exploring the attitudes toward new phenomena such as blockchain, since the relatively open-ended discussions can sensitize researchers to unrealized issues and hence increase the comprehensiveness of quantitative studies conducted afterward [29].

We recruited the participants for the focus group after the researchers’ university approved the research ethics proposal. We posted recruitment advertisements around the campus, a call for participants on the graduate student community online forum, and posts on social media platforms and online interest groups to recruit participants with diverse backgrounds. Since we aimed to maximize the diversity of participants, anyone with an interest in participating could participate regardless of their prior knowledge about blockchain. All participants signed a consent form approved by the research ethics office before the focus group discussions began.

A PhD student trained in archival science and human-centered design led the focus group discussions with the assistance of a master’s student of archival science. They began the focus groups by asking participants if they had negative experiences with the way health information is currently managed (questions 1.1 and 1.2 in Multimedia Appendix 1) and then explained to the participants the concept of blockchain and blockchain-based PHRs and solicitated participants’ intentions to adopt blockchain-based PHRs and their potential concerns (questions 2.1 to 2.6 in Multimedia Appendix 1). Notably, when explaining blockchain and blockchain-based PHRs, the researchers presented the participants a prototype of a user interface for a blockchain-based PHR, giving the participants a more concrete sense of blockchain and blockchain-based PHRs. Next, they asked the participants about their willingness to share health data with third parties and their concerns and attitudes toward health data sharing via blockchain, giving focused attention to topics frequently discussed in blockchain-based health data sharing, such as privacy, compensation, and wallet services (questions 3.1 to 5.1 in Multimedia Appendix 1).

### Sample Characteristics

In total, 26 individuals (13 men and 13 women) participated in our study: 8 in the first focus group, 8 in the second group, and 10 in the third group. A total of 22 participants were aged 25 to 35 years, 1 was aged 35 to 45 years, and 2 were aged 45 to 65 years. Among the participants, 3 participants had recently finished their advanced degrees (master’s or PhD) and the rest were enrolled in a master’s or PhD program. Five participants had an education background in information management or archival science, and 8 participants were enrolled in graduate programs in the medical field. All the participants had been patients at some point in their lives.

### Data Analysis

We performed the microinterlocutor analysis suggested by Onwuegbuzie et al [30]. The microinterlocutor analysis has been increasingly used to analyze focus group data in health-related research [31-33]. It not only reveals each participant’s attitude, stance, and arguments but also provides researchers with a quantitative overview of participant grouping [30]. Following Onwuegbuzie et al [30], we first read all the transcriptions of the focus group discussions, gaining an overall understanding of the transcriptions. Next, we coded participants’ responses to each discussion question separately for each focus group. As some participants diverged from the discussion, we paid attention to their words throughout the group discussion and coded their responses by interpreting all the words they contributed. By taking this step, we produced descriptive statistics for all the questions, as summarized in Multimedia Appendix 2. Multimedia Appendix 2 not only depicts how each participant responded to each question but also provides an overview of the responses of the group as a whole, based on which we generated the insights explained in the “Discussion” section. Finally, we imported the responses to each question into NVivo 12.0 (QSR International) and coded the thematic categories of explanations that participants provided for their responses, which helped us understand more deeply why the participants responded in certain ways. We used the thematic categories to structure our reporting on the open-ended questions, as seen in the “Results” section.

## Results

Multimedia Appendix 2 displays how each participant in the 3 focus groups responded to each question, including the indication of agreement, indication of dissent, ambivalent response, no response, and response given with an elaboration. In this section, we explain our results for each question, providing a descriptive statistical overview of the types of responses (including nonresponses) and qualitative categorizations of participants’ elaborations.

### Question 1.1: Health Data Distribution Without Consent

This question asked, “Can you recall hearing about any cases where someone’s personal health data were distributed without a patient’s consent?” In response, 9 participants indicated that they had heard of cases where patients’ health data were shared without consent, while 17 did not respond. Participants provided examples in which health data were involuntarily shared by others who sought profit, institutions attempting to control citizens, or health care professionals who were not vigilant in protecting the data. One participant described a story in which health care professionals secretly sold the health data of celebrities.

### Question 1.2: Interest in Controlling One’s Own Health Data

Participants were asked, “Are you interested in becoming the only controller of your own personal health data? Why or why not?” In total, 7 participants indicated that they were interested in becoming the only controller of their health data, 5 were not interested, 3 were ambivalent, and 11 did not respond. Those who were interested expressed that it is more convenient and morally legitimate for patients, especially those with chronic conditions, to control and profit from their data. As one participant said:

I have a chronic illness, and to get diagnosed I had to go see specialists, I had to have ultrasounds. I had to get blood tests, and to get the results of those I had to badger my receptionist at my doctor’s office to get her to give me the records, which are legally mine, and it was so frustrating because she made me feel like we shouldn’t even be asking. And so, I would really like to own my own records, because it feels very demoralizing when you can’t even have access to your own records.

Those who were not interested did not want to bear the risk of becoming the sole data owner, as they were worried about their inability to manage the data and the possibility of blocking data access when they were critically ill. Those who were ambivalent agreed with both sides.

### Question 2.1: Knowledge About Blockchain Technology

Participants were asked, “Do you have any questions about how blockchain technology works?” Prior to asking this question, the facilitator introduced the concept of distributed databases and their use in personal health data and compared blockchain to the centralized database technology currently used in health information management. Nine participants had questions about how blockchain works and 17 did not respond. Participants had questions about the management of private keys on blockchain, although researchers had explained that private keys are automatically generated by algorithms in tandem with public keys as pairs and that they are fundamentally different from passwords. Participants also had questions about where the private keys are stored and shared and how they might be managed, though the researchers had explained that the private key is not shared. That the participants asked those questions after the researchers had given detailed explanations suggests that participants had difficulty understanding the concept and features of private keys. Participants also asked questions related to the privacy and security of blockchain, including how they could revoke others’ access to their data on blockchain, how blockchain ensures the anonymity of transactions, and the proper use of health data. Notably, the issue of revoking access appeared throughout the focus group discussions.

### Question 2.2: Desire to Use Decentralized Databases

Participants were asked, “Would you consider using decentralized databases (blockchain) to control access to your health data? Would you consider using them to share your health data?” In total, 4 participants stated that they would use blockchain to control and share health data, 8 said they would not, 4 were ambivalent, and 10 did not respond. The reasons given by those who wanted to use blockchain included an affinity with the ideology of decentralized systems, perceived convenience for patients with chronic disease, and the security and privacy advantages of blockchain.

The reasons given by participants who did not want to use blockchain focused on the risk of losing private keys and hence losing access to all their data, which seemed to overshadow the perceived benefits. As one participant elaborated:

Because if I lose that private key my doctors cannot access my data. Even that company [third party] uses that data for promoting their devices. At the end, that’s to the benefit of patients.

Other reasons included distrust toward new technologies and worries about cyber attacks.

The ambivalent participants were mostly concerned about whether they could revoke access if they shared health data via blockchain and if the private key could be recovered by other security measures, for example, one’s biometrics.

### Question 2.3: Concerns About Privacy

We asked participants, “What concerns do you have about the privacy of your personal health data when using this technology, if any?” Eight participants expressed their concerns and others did not respond. Participants were concerned that blockchain may not truly protect privacy because bad actors can still print or take a screenshot of the shared data and disseminate them for profit. Participants were also concerned that they could not revoke access if they shared their health data with the wrong person, which breaches privacy.

### Question 2.4: Benefits of Blockchain in Ensuring Privacy

When asked, “Do you think there would be any benefits to using blockchain to ensure the privacy of your data?” 5 participants described the perceived benefits, while others did not respond. Participants perceived that the privacy ensured by blockchain could give rise to transparency, since one can see all the updates on one’s record. In addition, one could share the data with insurance companies to receive allowances.

### Question 2.5: Log-In Information

When asked, “Would you be willing to use a system that will secure your data, knowing that if you lost your log-in information you would not be able to recover it?” 7 participants stated they would not use it, since they could not recover the log-in information if lost; the rest did not respond. The participants were concerned that forgetting is part of human nature and that the private key is long. As one participant described:

I don’t have a good memory! [Laughter]

### Question 2.6: Password Recovery System

After the previous question, participants were asked, “If not, would you be willing to give up some of your individual control and security over your data in exchange for a password (private key) recovery system?” In total, 8 participants stated yes, 2 suggested no, and the rest did not respond. Those who stated yes suggested that the augmented control and security of a blockchain solution may actually hinder access to care, for example, if they need emergency care but forget their private key. They suggested that physicians could keep a copy of the private key or that third parties such as government or health authorities could use fingerprint or face recognition to recover the private key. One participant with a background in computer science who was unwilling to give up the security emphasized:

If you are giving up your security then you are weakening the system. So then it is no use to having that system.

Another participant who had also stated “no” stressed that the privacy of health data should never be compromised because some people may have a stigmatizing disease that they wish very few others to know about.

### Question 3.1: Sharing Information With Third Parties

Participants were asked, “Would you be willing to share your data with third parties (eg, universities, pharmaceutical companies, and private organizations) if they’re pseudonymous? Why or why not?” Three participants stated yes, 5 stated no, 4 were ambivalent, and 14 did not respond. One participant explained that he would share because sharing pseudonymous health data allows governments and researchers to gain new insights into diseases or public health while posing minimal risks to individual privacy. However, another participant explained that he would not share because the third party may be able to predict his identity even if the data were pseudonymous. Those who were ambivalent commonly suggested that if the purpose of the third party was virtuous (eg, informing public health decisions), they would be willing to share.

### Question 3.2: Trusted Third Party Types

When asked, “Which kinds of third parties would you be willing to share your data with?” 13 participants responded. Of these, 12 listed the third parties they would share the data with, and 1 expressed that he would not share the data with any third party. The third parties included universities, research institutions, government agencies, national health agencies, police and immigration officers, and pharmaceutical companies, which could be taxed for using the data. Among the third parties, universities and research institutions received the most support. As one participant suggested:

Universities are open. If not, it will come to a [drawback] to research. And in some cases you’ll probably have difficulties to discover new breakthroughs in sciences…so I wouldn’t mind sharing mine for research. We’re saving lives right? It’s all about saving lives.

Overall, participants indicated that they would share if the third party used the data for collective good rather than monetary gains. Participants commonly expressed unwillingness to share data with pharmaceutical companies, which were perceived to profit from people’s illnesses.

The participant who would not share data with any third party via blockchain argued that the third party may be able to identify him if they regularly receive his information.

### Question 3.3: Deciding Factors in Sharing Information With a Third Party

When asked, “What factors do you consider important when deciding to share your information with a third party?” 10 participants responded. The factors they listed included the end purpose of the third party (ie, whether the third party would use the data to make money or produce knowledge and therapeutics), whether the shared data had an expiry date, whether individuals could decide which data were shared, whether the third party could be audited when using the data, and whether the identity of the third party was the same as it claimed. The end purpose of the third party received the most emphasis.

### Question 3.4: Sharing Data Using Blockchain

Participants were asked, “Will you feel comfortable letting third parties (organizations, universities) see your data using blockchain? Do you think your data will be secure?” Only 2 participants responded to this question, and both indicated that they would share data via blockchain with a third party. One participant explained that he would only share the data when the third party was ethical and not corrupt.

### Question 4.1: Compensation for Sharing Personal Health Data

Participants were asked, “Would you seek compensation in exchange for securely sharing your personal health data?” Six participants indicated yes, 6 indicated no, and others did not respond. Those who would seek compensation believed that they should be compensated if the third party, especially pharmaceutical companies, were making money from their data. The reasons given by those who would not seek compensation included:

I only want to act from my heart.

As long as the third-party is producing knowledge, the knowledge is my compensation.

It is weird to commodify one’s body.

### Question 4.2. Important Factors in Fair Compensation

When asked, “If you were offered compensation in exchange for the use of your personal health data, what factors do you consider important when evaluating what makes for fair compensation for sharing your health data?” 9 participants responded. They suggested 3 factors that may influence the perceived fairness of the compensation: the amount of shared data relative to the amount of compensation, whether patients could receive free treatment, and the significance of the data relative to the amount of compensation. Participants indicated that if they shared DNA sequence or cell lines, they would ask for much more compensation.

### Question 4.3: Preferred Types of Compensation

We asked participants, “What type of compensation would you be looking for in exchange for your personal data?” A total of 12 participants responded to this question. They suggested the following types of compensation: free treatment, money, food, cryptocurrencies, discounts on health insurance, shared research findings, and donations to a good cause. Free treatment received the most mention, followed by cryptocurrencies and money.

### Question 5.1: Health Wallet Providers

Participants were asked, “Will your acceptance for the health data wallet service differ if it was provided by a company like Google? What about Apple? 23 and me? Facebook? A pharmaceutical company? Or an IT company like IBM?” Thirteen participants responded to this question. Among all the potential blockchain platform holders, IBM and government agencies received the most support, followed by Apple. Pharmaceutical companies, Google, and genetics companies received the most disapproval, as participants believed that Google already has too much information and pharmaceutical and genetics companies are ethically questionable.

## Discussion

### Key Findings

In this study, we investigated how health care consumers responded to the idea of blockchain-based PHRs and health data sharing through a focus group study with 26 individuals. Our primary findings are (1) consumers did not express stronger intentions to use blockchain-based PHRs and health data sharing when blockchain was introduced into the focus group discussion; (2) consumers were concerned about the risk of losing private keys, the revocability and expiration of data access, and the end purpose of the third party that could access their data via blockchain; and (3) consumers’ intentions may be improved if private key recovery is provided, governments or particular companies such as IBM or Apple provide the health wallet, the data shared via blockchain could expire and be revoked, and diverse types of compensation are provided for data sharing.

We started the focus group discussion by examining participants’ baseline attitudes toward self-ownership of health data (question 1.2) and found that 7 participants were interested and 7 were not interested, as shown in the first row of Multimedia Appendix 2. However, after blockchain was introduced as the enabling technology for PHRs (questions 2.1 and 2.2), the number of those who were interested decreased to 4 and the number of those who were not interested increased to 8, as shown in the fourth row of Multimedia Appendix 2. Our thematic coding suggests that although participants recognized the benefits of blockchain for augmented security and privacy, they were wary of PHRs enabled by blockchain, primarily due to the concern of forgetting or losing one’s private key. Interestingly, participants suggested that they were willing to give up the augmented security and privacy for the sake of emergency access to care (see the responses to question 2.6). Taken together, these findings suggest that consumers may not be substantially more interested (and might be less interested) in PHRs when presented with a blockchain-based application due to the concerns about losing one’s private key and the resulting inability to access care. These concerns complement the barriers to blockchain in health care, such as heightened transparency and low speed of transactions, that the literature is mainly focused on [1,13].

Even though consumers may not be inclined to take control of health records using blockchain, the responses to questions 2.6 and 5.1 suggest that certain design features may boost willingness. Specifically, the responses to question 2.6 suggest that patients may favor blockchain-based PHRs if key recovery is provided, although it may compromise the very advantage of blockchain. The response to question 5.1 suggests that patients would be more interested if the government, IBM, or Apple provided the health data wallet rather than Google, pharmaceutical companies, or genetic companies because the latter are perceived as ethically questionable or too powerful. Compared with the existing literature, in which design options have aimed to improve the technical features of blockchain, including consensus algorithms, oracle services, and data storage [1-4,13], the options provided here (ie, key recovery service and type of wallet provider) closely adhere to patients’ attitudes and behavioral patterns, indicating the importance of placing patients at the center of blockchain design.

We also investigated how consumers would respond to blockchain-based health data sharing. We started by asking their baseline willingness and found that it could be characterized as low (question 3.1). This result is different from prior literature, in which patients expressed high willingness to share health data with certain third parties [20,21]. However, since only 3 participants expressed a clear opinion in our study, the low willingness may not warrant an interpretation. After blockchain was introduced as the enabling technology (question 3.4), participants expressed roughly the same level of willingness (2 participants). This finding indicates that participants did not become more interested in health data sharing when presented with blockchain, which provides more privacy and security.

The responses to questions 3.2 and 3.3 can explain this response. Similar to prior studies [20,21], participants’ willingness to share data was mostly affected by factors such as the end purpose of the third party (ie, making money or developing knowledge) and whether the third party would be audited. These factors pertain to the characteristics of the third party, over which the existing blockchain technology still has little control. Admittedly, blockchain technology has upgraded significantly in the past decade [34]; however, blockchain-based solutions to enforce patient-stipulated data use policies have yet to emerge. Other factors, such as the chance to revoke access or impose an expiration date on consent for use, also contributed to users’ reluctance to rely on blockchain, as these were perceived as being currently unavailable capabilities of blockchain, though blockchain system designers have recently developed solutions to revoke access [35]. This implies that for consumers to be more willing to share health data via blockchain, not only must blockchain system designers reconfigure blockchain so that patients can revoke access or designate an expiration time for data access but, more importantly, consumers need to be educated about the technical updates and novel capabilities of this still emerging technology.

Other measures to improve consumers’ willingness may include diversifying the types of compensation for sharing data and providing nonmonetary compensation, such as free treatment, shared research results, and donations to a good cause (see the responses to questions 4.1 to 4.3). Although value tokens or cryptocurrencies are popular types of compensation on blockchain-based applications to incentivize data sharing, the participants in our study placed greater value on nonmonetary forms of compensation. This finding expands the notion of health information altruists [36] by suggesting that altruists are not only willing to share data for altruistic reasons, such as helping medical research, but are also drawn to altruistic compensation forms. Overall, these design options highlight the benefits of the principle of patient-centered design [37,38], inviting a shift from improving technical properties of blockchain to understanding consumers’ attitudes and needs.

### Limitations

This study has a few limitations. First, the sample size is relatively small and not demographically diverse. Most of the participants were younger and held or were pursuing an advanced degree. This composition of the sample means our findings are conservative because young people with advanced degrees tend to be more technology savvy than older, less educated adults; if their intentions for adoption are low, the intentions of the broader population would be even lower. As such, we suggest that a more diverse sample would not weaken, and could even strengthen, our results. Nevertheless, future studies may recruit participants with more diverse backgrounds to approximate the average intention of the broader population. A related limitation is that many participants did not respond to our questions during the group discussion. Although nonresponses are not uncommon in focus groups [29,30], the nonresponses reduced our capacity to use the sample. Future research may recruit more participants or conduct more focus groups to collect more responses. Second, although the study participants had patient experience and could be considered patients in a broad sense, the study was not conducted in a clinical setting where the participants are ill and faced with the immediate task of managing their health records or sharing their health data with clinicians. This limitation may not be significant for the time being, since researchers have only begun to understand individuals’ intentions to adopt blockchain-based health care applications and there is still little evidence that real-time disease experiences would affect consumers’ intentions to use this technology. Future research may explore whether and in what way real-time disease experiences affect intentions to adopt blockchain-based PHRs; these studies should be conducted with patients in a clinical setting to explore how their intentions would differ from those reported in this paper. Third, since blockchain technology evolves quickly, the participants were not aware of the most recent capabilities of the technology. Future studies may conduct field experiments, allowing a large group of participants to make decisions on whether to own and share health data using an actual blockchain application that is equipped with the most recent technical developments of blockchain, and compare the findings with this study’s findings. Fourth, although the findings from this study have implications for designing user-centered blockchain PHR and data-sharing applications, it is focused on exploring individuals’ intentions and concerns rather than providing a comprehensive list of design recommendations. Based on the findings of this study, future research may put a stronger focus on user-centered design and describe detailed design features. Finally, only 1 participant in our sample indicated that he had prior knowledge about blockchain. Thus, we do not have enough evidence to discern whether prior knowledge about blockchain would affect individuals’ intentions to adopt blockchain-based PHRs and health data sharing. We can only suggest that the effect may be ambiguous, since individuals can have weaker intentions to adopt blockchain-based PHRs if their prior knowledge about blockchain is that blockchain is associated with scammer cryptocurrency or stronger intentions if they understand and their knowledge is thorough and updated. As such, future research may systematically investigate the effect of prior knowledge of blockchain on the intention to adopt blockchain-based health care applications.

In conclusion, our study did not show strong intentions among health care consumers to adopt blockchain-based PHRs and heath data sharing, and the exploration of consumers’ concerns and preferences revealed several design options that may increase the adoption intention. As understanding consumer attitudes can contribute to the dynamic coevolution of technical capabilities and user behaviors and hence enhance the development and adoption of blockchain in health care, our study lays the stepping stone for patients and blockchain system designers to become cocreators of blockchain-based health information systems so that health and patient privacy can be simultaneously optimized. This may prove critical to tackling pandemics such as COVID-19, during which public health depends on patient health data privacy and sharing.

## Appendix

Multimedia Appendix 1 Focus group question list.

Multimedia Appendix 2 Microinterlocutor analysis.

## Abbreviations

PHR: personal health record
