# Supplementary material for: Consumers’ Intentions to Adopt Blockchain-Based Personal Health Records and Data Sharing: Focus Group Study
Source: JMIR Form Res. 2020 Nov 5;4(11):e21995. doi: 10.2196/21995 (PMC7677023; doi:10.2196/21995)
Supplement: Multimedia Appendix 1 [file formative_v4i11e21995_app1.docx]

**Appendix 1 Focus Groups Question List**

**Set 1: Health Data Management**

1.1 Can you recall hearing about any cases where someone’s personal health data was distributed without a patients’ consent?

1.2 Are you interested in becoming the only controller of your own personal health data? Why or why not?

(i.e. you are the only one who could for example send your blood test result to your family doctor, or walk-in clinic doctor.)

**Set 2: Distributed databases and Blockchain technology**

2.1 Do you have any questions about how Blockchain technology works?

2.2 Would you consider using decentralized databases (blockchain) to control access to your health data?

Would you consider it to share your health data?

2.3 What concerns do you have about the privacy of your personal health data when using this technology? If any?

2.4 Do you think there would be any benefits to using Blockchain

to ensure the privacy of your data?

2.5 Would you be willing to use a system that will secure your data, knowing that if you lost your login information you would not be able to recover it?

2.6 If no, would you be willing to give up some of your individual control and security over your data in exchange for a password recovery system?

**Set 3 : Sharing health data**

3.1 Would you be willing to share your data with 3rd parties if it’s pseudo anonymous (e.g. universities, pharmaceutical companies, and private organizations) ? Why and why not?

3.2 Which kind of 3rd parties you would be willing to share it with?

3.3 What factors do you consider important when deciding to share your information with a 3rd party?

3.4 Will you feel comfortable letting 3rd parties (organizations, universities) see your data using blockchain? Do you think it will be secure?

**Set 4: Compensation for Sharing Data**

4.1 Would you seek compensation in exchange for securely sharing your personal health data?

4.2 If you were offered compensation in exchange for the use your personal health data, what factors do you consider important when evaluating what makes for fair compensation for sharing your health data?

4.3 What type of compensation would you be looking for in exchange for your personal data?

Follow up if they are clueless: Discounts on potential goods and services (coupons), other?

**Set 5:**

5.1 Will your acceptance for the health data wallet service differs if it was provided by a company like Google. What about Apple? 23 and me? Facebook? A pharmaceutical company? Or an IT company like IBM?
