# Supplementary material for: Consumers’ Intentions to Adopt Blockchain-Based Personal Health Records and Data Sharing: Focus Group Study
Source: JMIR Form Res. 2020 Nov 5;4(11):e21995. doi: 10.2196/21995 (PMC7677023; doi:10.2196/21995)
Supplement: Multimedia Appendix 2 [file formative_v4i11e21995_app2.docx]

**Table S1.** Microinterlocutor analysis.

| **Question** | **Participant No.** | | |
| --- | --- | --- | --- |
|  | **Group 1** | **Group 2** | **Group 3** |

|  | **1** | **2** | **3** | **4** | **5** | **6** | **7** | **8** | **9** | **10** | **11** | **12** | **13** | **14** | **15** | **16** | **17** | **18** | **19** | **20** | **21** | **22** | **23** | **24** | **25** | **26** |
| --- | --- | --- | --- | --- | --- | --- | --- | --- | --- | --- | --- | --- | --- | --- | --- | --- | --- | --- | --- | --- | --- | --- | --- | --- | --- | --- |
| 1.1 | A^a^ | SE^b^ | NR^c^ | NR | NR | NR | NR | SE | SE | NR | NR | NR | NR | NR | SE | SE | NR | NR | NR | SE | NR | NR | NR | SE | NR | SE |
| 1.2 | A | NR | A | A | A | D^d^ | NR | D | SE | SD^e^ | NR | NR | NR | NR | SE | NR | SE | NR | AR^f^ | NR | NR | AR | NR | SD | AR | SD |
| 2.1 | SE | NR | NR | NR | NR | NR | NR | SE | SE | SE | SE | NR | SE | NR | NR | SE | NR | NR | SE | NR | SE | SE | SE | NR | NR | NR |
| 2.2 | NR | SD | SE | NR | SE | SE | NR | SD | SE | NR | SD | SD | SD | AR | SD | SD | NR | NR | AR | SD | NR | AR | AR | NR | NR | NR |
| 2.3 | NR | NR | SE | NR | NR | NR | NR | NR | SE | NR | NR | NR | NR | NR | NR | SE | SE | NR | SE | NR | SE | SE | SE | SE | NR | NR |
| 2.4 | SE | NR | SE | NR | NR | NR | NR | NR | NR | NR | NR | NR | NR | NR | NR | NR | SE | NR | NR | NR | NR | SE | NR | NR | SE | NR |
| 2.5 | NR | NR | SD | SD | NR | SD | NR | NR | SD | NR | NR | NR | NR | NR | NR | NR | SD | NR | SD | NR | NR | NR | NR | SD | SD | NR |
| 2.6 | A | NR | NR | NR | NR | SE | A | NR | SE | NR | NR | NR | SE | NR | SE | SD | NR | NR | NR | SD | NR | NR | SE | SE | NR | NR |
| 3.1 | D | D | D | D | NR | NR | NR | NR | AR | SD | NR | A | NR | AR | NR | AR | NR | NR | NR | AR | NR | NR | A | NR | SE | NR |
| 3.2 | SE | SE | SE | SE | SE | SE | SE | SD | SE | SE | NR | NR | SE | NR | NR | NR | SE | NR | NR | NR | NR | NR | NR | SE | NR | NR |
| 3.3 | NR | NR | NR | SE | SE | NR | SE | SE | NR | SE | SE | NR | NR | SE | NR | SE | NR | NR | NR | NR | NR | SE | NR | SE | NR | NR |
| 3.4 | AR | NR | NR | NR | NR | A | NR | NR | NR | NR | NR | NR | NR | NR | NR | NR | NR | NR | NR | NR | NR | NR | NR | NR | NR | NR |
| 4.1 | SE | SD | SE | SE | NR | NR | NR | NR | NR | SD | NR | SE | NR | NR | SD | SE | NR | NR | NR | SD | NR | SE | SD | SD | NR | NR |
| 4.2 | SE | SE | SE | NR | SE | SE | NR | SE | SE | NR | NR | SE | NR | NR | NR | SE | SE | NR | NR | NR | NR | NR | NR | NR | NR | NR |
| 4.3 | SE | NR | SE | NR | SE | SE | NR | SE | SE | NR | SE | SE | NR | NR | NR | SE | NR | NR | NR | NR | SE | SE | NR | SE | NR | NR |
| 5.1 | NR | NR | NR | NR | NR | NR | NR | NR | SE | SE | SE | SE | NR | NR | NR | SE | SE | NR | SE | SE | SE | SE | SE | SE | SE | NR |

^a^A: indicated agreement (verbal or nonverbal).

^b^SE: Provided significant statement or example suggesting agreement.

^c^NR: Did not indicate agreement or dissent (ie, nonresponse or did not know).

^d^D: Indicated dissent (verbal or nonverbal).

^e^SD: Provided significant statement or example suggesting agreement.

^f^AR: ambivalent response.
